# Effect of MgO and Fe_2_O_3_ dual sintering aids on the microstructure and electrochemical performance of the solid state Gd_0.2_Ce_0.8_O_2-δ_ electrolyte in intermediate-temperature solid oxide fuel cells

**DOI:** 10.3389/fchem.2022.991922

**Published:** 2022-09-27

**Authors:** Qingwen Liang, Ping Tang, Jing Zhou, Jinghe Bai, Dan Tian, Xiaofei Zhu, Defeng Zhou, Ning Wang, Wenfu Yan

**Affiliations:** ^1^ School of Chemistry and Life Science, Changchun University of Technology, Changchun, China; ^2^ Shenzhen Institute of Advanced Technology, Chinese Academy of Sciences, Shenzhen, China; ^3^ College of Materials Science and Engineering, Nanjing Forestry University, Nanjing, China; ^4^ Shenzhen Institute of Advanced Electronic Materials, Shenzhen, China; ^5^ State Key Laboratory of Inorganic Synthesis and Preparative Chemistry, College of Chemistry, Jilin University, Changchun, China

**Keywords:** solid oxide fuel cells, sintering aid, electrolyte, Gd_0.2_Ce_0.8_O_2-δ_ (GDC), ionic conductivity

## Abstract

Solid state electrolytes have been intensively studied in the solid oxide fuel cells (SOFCs). The aim of this work is to investigate the effects of MgO and Fe_2_O_3_ dual sintering aids on the microstructure and electrochemical properties of solid state Gd_0.2_Ce_0.8_O_2-δ_ (GDC) electrolytes, which are prepared by a sol-gel method with MgO and Fe_2_O_3_ addition to the GDC system. It is found that the addition of MgO and Fe_2_O_3_ can reduce the sintering temperature, increase densification and decrease the grain boundary resistance of the electrolyte. The 2 mol% MgO and 2 mol% Fe_2_O_3_ co-doped GDC (GDC-MF) exhibits the highest grain boundary conductivity. At 400°C, the grain boundary conductivity and total conductivity of GDC-MF are 15.89 times and 5.56 times higher than those of GDC. The oxygen reduction reaction (ORR) rate at the electrolyte/cathode interface of GDC-MF is 47 % higher than that of GDC. Furthermore, the peak power density of a single cell supported by GDC-MF is 0.45 W cm^−2^ at 700°C, 36.7% higher than that of GDC. Therefore, the GDC-MF should be a promising electrolyte material for intermediate-temperature solid oxide fuel cells (IT-SOFCs).

## 1 Introduction

As the critical components of solid oxide fuel cells (SOFCs), solid electrolytes have the advantages of high ionic conductivity, high density, and high chemical compatibility (Santos et al., 2018; Spiridigliozzi et al., 2019; Singh et al., 2021). Electrolytes usually require high sintering temperatures (≥1400°C) to obtain sufficiently high densities and electrical conductivities (Wang et al., 2019). However, the high sintering temperature will lead to a series of challenges, e.g., high manufacturing cost, fast aging rate of the cell, and the poor compatibility of the components. Therefore, it is of great significance to develop new solid electrolytes with high ionic conductivity in the intermediate-temperature (500–700°C) range.

CeO_2_-based solid electrolytes were the widely investigated IT-SOFCs electrolytes because of the high ionic conductivity at low/medium temperature and excellent compatibility with the commonly used electrode materials (Coduri et al., 2018; Zhang et al., 2021). A small amount of low melting point additives were often added to CeO_2_-based solid electrolyte as sintering aids during the process of sintering, e.g., Fe_2_O_3_ (Xu et al., 2011; Zheng et al., 2011; Mehranjani et al., 2017), MgO(Cho et al., 2007; Li et al., 2008; Bi et al., 2017), ZnO (Nicollet et al., 2017), and Bi_2_O_3_ (Accardo et al., 2018) etc. This method can significantly reduce the sintering temperature and improve the density of the electrolyte. Fe_2_O_3_ and MgO have been reported to be the excellent sintering aids for improving the ionic conductivity and relative density of Gd_0.2_Ce_0.8_O_2-δ_ (GDC) electrolyte (Zheng et al., 2011; Bi et al., 2017; Shajahan et al., 2020). Zheng et al. added 0.25 mol% Fe_2_O_3_ to Ce_0.8_Sm_0.2_O_1.9_ electrolyte and obtained the improved densification and excellent total conductivity (σ_t_) (Zheng et al., 2011). Similarly, Mehranjani et al. reported that with 2 mol% Fe_2_O_3_ as sintering aids, yttria-stabilized zirconia (YSZ) and GDC bilayer electrolytes could exhibit the reduced sintering temperature from 1450 to 1300°C and the improved densification (Mehranjani et al., 2017). Moreover, the liquid-phase sintering mechanism of Fe_2_O_3_ gave rise to the good continuity and permeability at the GDC grain boundary, which effectively promoted the migration of oxygen ions (Zhang et al., 2004a). In addition, MgO is another effective sintering aid, which can simultaneously improve the density and conductivity of the electrolyte. For instance, adding 1.0 mol% MgO to GDC electrolyte as sintering aids could reach the relative densities of 95% after sintering at 1200°C for 10 h (Bi et al., 2017). Molten MgO can promote GDC grain growth and the degree of densification of the sample. Moreover, the difference of the ionic radii between Mg^2+^ (0.89 Å) and Ce^4+^ (0.97 Å) will arouse the lattice distortion, which helps grain boundary movement and grain growth during the sintering process. Additionally, the solid solubility of Mg^2+^ in the CeO_2_ lattice was as low as ∼1 mol% (Cho et al., 2007), when the amount of Mg^2+^ exceeds the solubility, the redundant MgO will populate at the grain boundaries. Therefore, the addition of MgO can optimize the structure of the space charge layer and remove the silicon phase at the grain boundaries, improving the overall ionic conductivity (Li et al., 2008).

In recent years, intense research has been focused on the double-sintering aids strategy on ceria-based electrolytes (Jia et al., 2015; Chen et al., 2020; Babar et al., 2022). For example, the Nd_0.2_Ce_0.8_O_2-δ_ (NDC) solid electrolyte with CoO-Bi_2_O_3_ (molar ratio of 1:1) dual sintering aids achieved the density of 95.3 % after sintered at 1100°C for 10 h (Chen et al., 2020). At 800°C, the σ_t_ was 5.765 × 10^−2^ S cm^−1^, which was higher than that of the NDC electrolytes added with single sintering aids CoO (3.802 × 10^−2^ S cm^−1^) or Bi_2_O_3_ (4.649 × 10^−2^ S cm^−1^). Similarly, the electrochemical property and sintering behavior of the Ce_0.8_Y_0.2-x_Cu_x_O_2-δ_ electrolytes were improved by adjusting the Y/Cu ratio (Jia et al., 2015). Ce_0.8_Y_0.2-x_Cu_x_O_2-δ_ electrolyte sintered at 1300°C for 4 h could achieve a high relative density of 95%. It can be seen that selecting a suitable sintering aid is an important approach to improve the conductivity of the ceria-based electrolyte. Compared with single sintering aids, adding dual sintering aids is also an effective strategy to improve material densification. The addition of MgO or Fe_2_O_3_ can form liquid phase sintering and promote electrolyte densification at low temperatures.

Usually the doping amount of the sintering aid is less than 5 mol% (Zhang et al., 2017; Han et al., 2018). With this in mind, in this paper, the addition of 4 mol% MgO-Fe_2_O_3_ dual sintering aids were proposed to promote the electrochemical performance of Gd_0.2_Ce_0.8_O_2-δ_ solid state electrolyte in the IT-SOFCs.

## 2 Experiment methodology

### 2.1 Chemicals

The chemicals in this paper including Ce(NO_3_)_3_·6H_2_O (99.99%), Gd(NO_3_)_3_·6H_2_O (99.99%), Mg(NO_3_)_2_·6H_2_O (99%), Fe(NO_3_)_3_·9H_2_O (99%), La(NO_3_)_3_·6H_2_O (99.9%), Sr(NO_3_)_2_ (99%), Fe(NO_3_)_3_·9H_2_O (99.99%), Co(NO_3_)_2_·6H_2_O (99.99%), glycine (99%), NiO (99.9%), Polyethylene glycol (PEG 2000) and solid citric acid (C_6_H_8_O_7_·H_2_O, 99.5%), were purchased from Aladdin. Those chemicals were used as received without further purification.

### 2.2 Material preparation and cell fabrication

#### 2.2.1 GDC-based electrolytes preparation

GDC and GDC-based electrolyte powders with MgO and Fe_2_O_3_ sintering aids were prepared by sol-gel method. According to the stoichiometric ratio, Ce(NO_3_)_3_·6H_2_O, Gd(NO_3_)_3_·6H_2_O, Mg(NO_3_)_2_·6H_2_O and Fe(NO_3_)_3_·9H_2_O were accurately weighed and dissolved in distilled water, orderly. Polyethylene glycol and solid citric acid weighed 1.5 times of metal ions were added to the solution under continuous stirring at the room temperature to obtain the precursor solution of GDC. Then, the solution was dehydrated at 75°C to obtain a dry gel, and calcined in a muffle furnace at a heating rate of 5°C min^−1^ to 650 °C for 6 h to obtain GDC and GDC-based electrolyte powders with different proportions of MgO and Fe_2_O_3_ sintering aids. Subsequently, after all powder samples were thoroughly ground in an agate mortar, the GDC samples with 4 mol% MgO, 4 mol% Fe_2_O_3_, 2 mol% MgO+2 mol% Fe_2_O_3_ added were labeled as GDC-M, GDC-F and GDC-MF, respectively. In order to obtain samples with similar thickness, ∼0.5 g of different electrolyte powders were weighed and pressed into discs with a diameter of 13 mm and a thickness of 1.2 mm under the pressure 10 MPa. Then, all pressed samples were sintered in a muffle furnace at 1200°C for 10 h at a heating rate of 5°C min^−1^ to obtain dense electrolyte sheets. Finally, both sides of the electrolyte sheets were coated with silver paste and heated at 700°C for 30 min for electrochemical performance test after polishing and ultrasonic cleaning.

#### 2.2.2 Electrodes preparation

La_0.6_Sr_0.4_Co_0.8_Fe_0.2_O_3-δ_ (LSCF) cathode powder was prepared by glycine-nitrate method (Bai et al., 2020). According to the stoichiometric ratio, La(NO_3_)_3_·6H_2_O, Sr(NO_3_)_2_, Fe(NO_3_)_3_·9H_2_O and Co(NO_3_)_2_·6H_2_O were accurately weighed and dissolved in distilled water, orderly. Glycine was added to the solution at the ratio of 1.5 times higher than the $NO_{3}^{-}$ under continuous stirring at 30°C. The mixture was heated and stirred at 110°C for combustion. After the combustion was completed, the product was calcined at 750°C for 4 h at a heating rate of 5°C min^−1^ in a muffle furnace to obtain LSCF cathode power.

The GDC and GDC-MF electrolyte powders were mixed with LSCF cathode and NiO anode (mass fraction ratio of 50:50) in ethanol, respectively, and then calcined in air at 950°C for 15 h to evaluate the SOFC performance and compatibility with the electrolyte.

#### 2.2.3 Cell fabrication

The symmetrical cells and single-cells were prepared to investigate the electrochemical behavior of fuel cells. The electrode powders were homogeneously mixed with 3 wt% terpineol/ethylene cellulose and fully ground in an agate mortar for 30 min to produce the cathode slurry. The LSCF cathode slurry was symmetrically coated on both sides of the GDC-MF and GDC pellets using screen printing technique. The effective area and thickness of the cathode were controlled to be ∼0.25 cm^2^ and ∼20 μm, respectively, and calcined at 950 °C for 2 h. The Ag slurry was applied to the grid structure on both sides of the cell as the current collector, and then dried at 100°C for 2 h to obtain a symmetrical cell.

Both sides of the GDC and GDC-MF electrolyte sheets were polished to a thickness of ∼300 μm and placed in ethanol for ultrasonic cleaning. The NiO-GDC (percentage ratio of 6:4) anode were prepared by ball milling, and 6 wt% terpineol/ethylene cellulose was added to form the anode slurry. The anode slurry was screen-printed on one side of the GDC-MF and GDC pellets and calcined at 1250°C for 4 h. The cathode ink was coated on the other side of the electrolyte pellets and calcined at 950°C for 2 h. The effective area and thickness of the electrode were controlled to be consistent with the symmetric cells. The anode of the fuel cell was attached and sealed to one end of the quartz tube by using silver paste.

### 2.3 Characterization

The X-ray diffractometer (XRD, Rigaku D/MAX-2000/PC, Japan) equipped with Cu Kα_1_ radiation (*λ* = 0.15406 nm, 40 kV and 40 mA; scanning rate: 4° min^−1^) was used to characterize the crystal structure and compatibility of the sintered samples. The Raman spectrometer (Jobin-Yvon Horiba Scientific) with 532 nm excitation wavelength was carried out to analyze oxygen vacancy concentration of powders from 100 cm^−1^ to 1000 cm^−1^. Surface chemistry analysis of the GDC-based electrolyte powders were identified by X-ray Photoelectron Spectroscopy (XPS). The microstructure of electrolyte sheets was determined by field emission scanning electron microscopy (FE-SEM, SUPPA 40, ZEISS, Germany). The thermal expansion coefficient (TEC) of the sintered samples was characterized by thermal expansion analyzer (Netzsch DIL 402C) in the range of 50–850°C (heating rate: 3°C min^−1^; standard sample: Al_2_O_3_). The relative densities of all sintered samples were tested by the Archimedes method and calculated by Eq. 1:

$$\rho_{\mathrm{rel}}=\frac{\rho_{A}}{\rho_{T}}\times \mathrm{100}\%$$ (1)

where ρ_A_ is the actual density measured using Archimedes’ principle, ρ_T_ is the theoretical density of the sintered sample obtained from X-ray diffraction data using Jade software, and ρ_rel_ is the relative density of the sintered sample.

The grain size of the sintered samples was calculated according to the following Debye Scherrer formula (Eq. 2):

$$d_{\mathrm{XRD}}=\frac{\mathrm{k\lambda}}{\mathrm{\beta cos\theta}}$$ (2)

where d_XRD_、K、λ、θ and β are the crystalline size, Scherrer constant (0.89), X-ray wavelength (0.154,056 nm), Bragg diffraction angle and diffraction peak half-width, respectively.

### 2.4 Electrochemical performance

The Electrochemical Impedance Spectroscopy (EIS) was carried out on a Solartron 1260–1287 workstation under the following conditions: open-circuit voltage (OCV), AC perturbation voltage of 10 mV, frequency range of 100 KHz-0.1 Hz, temperature range of 300–800°C and temperature interval of 50°C. The EIS data was analyzed and fitted by Ziew software. The conductivity of the samples was calculated according to the following Eq. 3:

$$\mathrm{\sigma =}\frac{D}{\mathrm{RS}}$$ (3)

in which D, S and σ correspond to the thickness, surface area and conductivity of the sample respectively.

## 3 Results and discussion

### 3.1 Microstructural analysis

#### 3.1.1 Crystalline phase analysis

**FIGURE 1:**
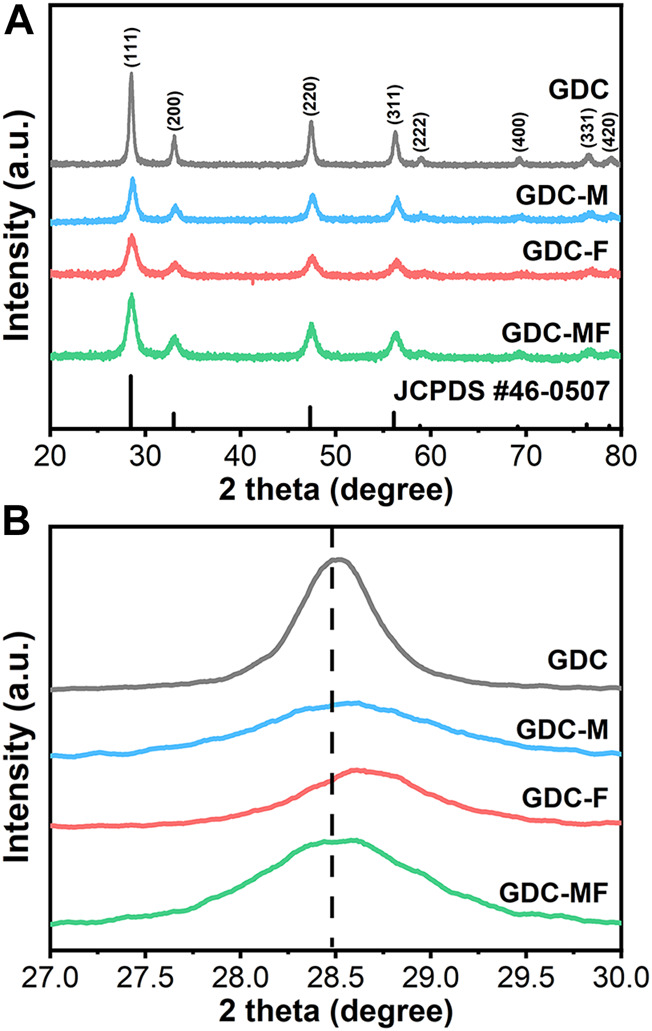
**(A)** X-ray diffraction (XRD) patterns and **(B)** Zoom-in XRD patterns in a 2θ range of 27.00–30.00° of GDC-based electrolytes.

In order to determine the effect of the addition of MgO and Fe_2_O_3_ on the structure of GDC electrolyte, the XRD spectra of all samples are shown in Figure 1, and the unit cell parameters of the samples are listed in Table 1. As can be seen from Figure 1A, the diffraction peaks of all samples correspond to the characteristic peaks of the cubic fluorite structure (JCPDS #46–0507) of GDC, and no additional impurity peaks were observed. To further clarify the effect of MgO and Fe_2_O_3_ addition on GDC structure, the diffraction peak (111) of the sample was amplified (2θ = 27.0–30.0°) and shown in Figure 1B. Combined with Figure 1B and Table 1, it can be seen that the diffraction peak (111) of GDC and GDC-M samples have almost no deviation. Moreover, the unit cell parameters of GDC and GDC-M were also almost unchanged, this could be discussed from the following two aspects. On the one hand, Mg^2+^ entering the GDC lattice would lead to a decrease in unit cell parameters due to the radius of Mg^2+^ (0.89 Å) is smaller than that of Ce^4+^ (0.97 Å); On the other hand, it is extremely low of the solid solubility of MgO (∼1 mol%) in GDC. When the solid solubility is exceeded, the remaining MgO will remain at the grain boundaries, which increases the unit cell parameters. In addition, compared with the diffraction peak (111) of GDC, the diffraction peak of GDC-F was slightly shifted toward the high diffraction angles, indicating that the addition of Fe_2_O_3_ could reduce the cell volume of GDC (Table 1). This result could be attributed to the solid solubility of Fe_2_O_3_ in GDC more than 4 mol% (Zheng et al., 2011; Abbas et al., 2015). Therefore, Fe^3+^ (0.78 Å) with smaller radius could easily enter the GDC lattice to replace Ce^4+^ (0.97 Å) ions, resulting in the shrinkage of the unit cell volume. It is worth noting that the diffraction peak of GDC-MF (111) was shifted to the high diffraction angles less than that of GDC-F (Figure 1B), and the unit cell parameters was also similar to GDC-M sample. This is mainly attributed to the co-addition of 2 mol% Fe_2_O_3_ and 2 mol% MgO which increases the lattice parameters of the GDC samples as a result of phase equilibrium.

**TABLE 1:** Relevant parameters of GDC-based electrolytes sintered at 1200°C for 10 h.

Samples	a (Å)	Vol (Å^3^)	ρ_T_ (g·cm^−3^)	ρ_a_ (g·cm^−3^)	ρ_Rel_ (%)	d (nm)
GDC	5.4139	158.68	7.249	6.301	86.9	14.90
GDC-M	5.4140	158.70	7.254	6.761	93.2	15.27
GDC-F	5.4115	158.47	7.253	6.898	95.1	18.35
GDC-MF	5.4135	158.66	7.257	7.228	96.6	25.39

a: cell parameters; Vol: unit cell volume; ρ_T_: theoretical density; ρ_a_: actual density; ρ_Rel_: relative density; d: crystallite size.

#### 3.1.2 Raman spectroscopy

**FIGURE 2:**
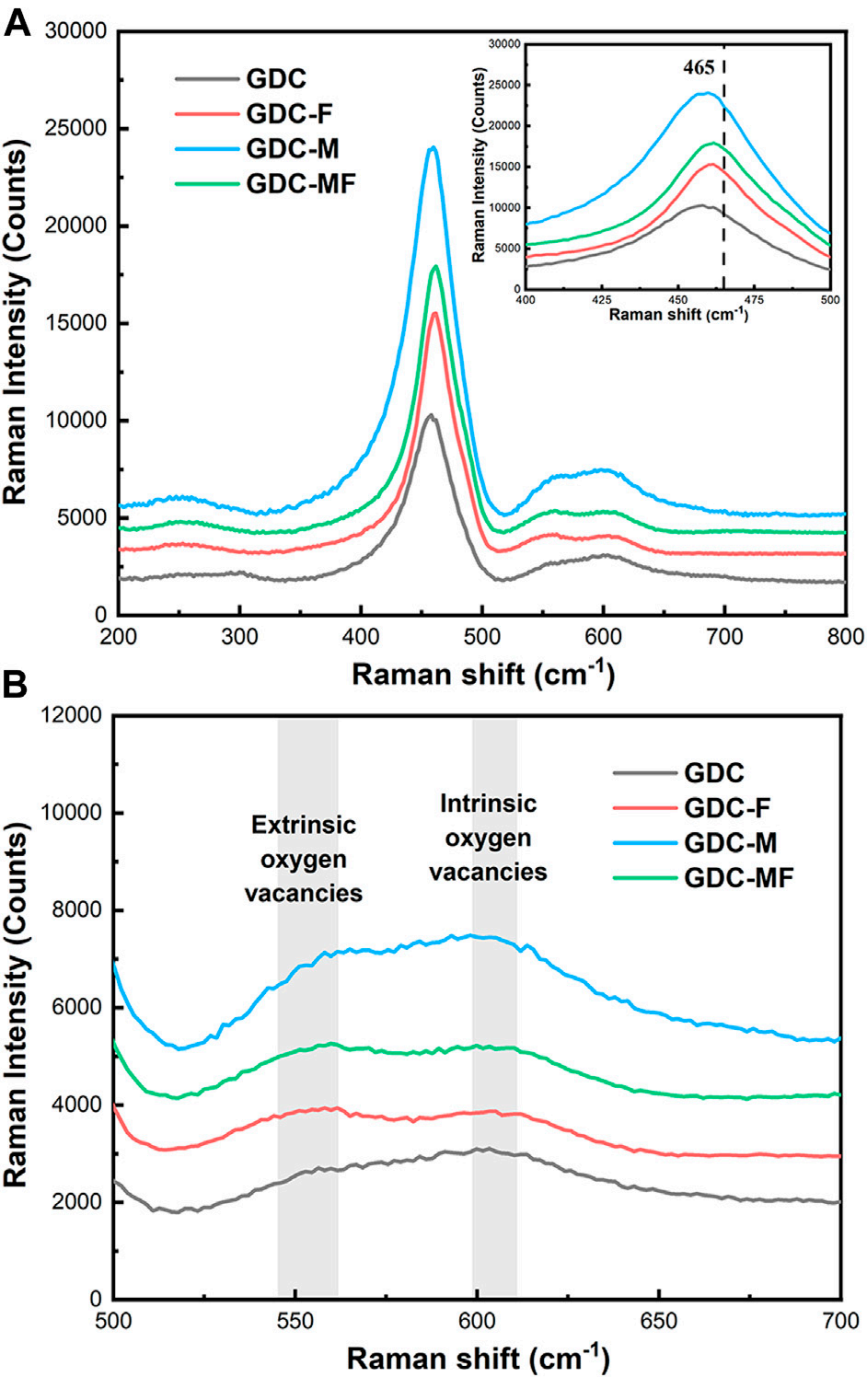
**(A)** Raman spectra of all samples, inset is the magnified F_2g_ band and **(B)** Oxygen vacancy pattern.

The effect of MgO and Fe_2_O_3_ additions on the GDC structure and new defect sites were tested by the Raman spectrometer at room temperature, as shown in Figure 2A. From the Raman spectrum, all samples had a weak absorption band at 245 cm^−1^, which could be attributed to a double degenerate transverse optical mode (Li et al., 2009). In addition, the Raman peak around 465 cm^−1^ was associated with the F_2g_ vibrational mode of the cubic lattice, indicating that the CeO_2_-based electrolytes still maintain the perfect cubic fluorite structure (Nakajima et al., 1994; Li et al., 2020; Song et al., 2021), which was consistent with the XRD results. Moreover, the positions of the vibrational peaks around 465 cm^−1^ for GDC-M and GDC samples were consistent, basically. However, for GDC-F and GDC-MF samples, the vibrational peaks around 465 cm^−1^ were slightly shifted toward higher frequencies and slightly broadened, which may be related to the formation of solid solutions (Accardo et al., 2021). The shift of the vibrational peak suggests that the addition of Fe_2_O_3_ to CeO_2_ might form a solid solution and this difference might be related to the low solubility of MgO in GDC (∼1 mol%). In addition, the defects caused by doping could also interfere with the signal at ∼465 cm^−1^ (Sartoretti et al., 2019), suggesting that there might be dopant ions entering the CeO_2_ lattice to replace Ce^4+^ to generate oxygen vacancies.

The locally enlarged Raman spectra was shown in Figure 2B. Characteristic absorption peaks associated with the vibration of oxygen vacancies in the defect space were observed at ∼550 and ∼600 cm^−1^ (Anjaneya et al., 2014; Song et al., 2021). Among them, the signal at ∼550 cm^−1^ was attributed to the vibration of oxygen vacancies in the defect space generated by the introduction of low-valent cations into the CeO_2_ lattice (Guo and Waser, 2006). With the addition of sintering aids MgO and Fe_2_O_3_, the signal intensities of GDC-F, GDC-M and GDC-MF were stronger than that of GDC at 550 cm^−1^ (Figure 2B). This was because the small amount of Mg^2+^ or Fe^3+^ enters the CeO_2_ lattice to replace Ce^4+^ to generate oxygen vacancies, resulting in the enhanced signal intensity at 550 cm^−1^. The corresponding defect equations are shown in Eqs 4, 5:

$$Fe_{2}O_{3}\overset{\mathrm{Ce}O_{2}}{\to }\mathrm{2F}e_{\mathrm{Ce}}^{\prime }+{\ddot{V}}_{O}\mathrm{+2}O_{O}$$ (4)

$$\mathrm{MgO}\overset{\mathrm{Ce}O_{2}}{\to }Mg_{\mathrm{Ce}}^{\prime \prime }+{\ddot{V}}_{O}+O_{O}$$ (5)

Since the signal at 550 cm^−1^ was related to the oxygen vacancy, the area ratio of this peak to the peak of 464 cm^−1^, i.e., *A*550/*A*464, could estimate the oxygen vacancy concentration (Pu et al., 2007). Compared to the GDC (0.0279) sample, the oxygen vacancy concentrations of GDC-F (0.0649), GDC-M (0.0367) and GDC-MF (0.0584) were all increased. This result confirms that the Ce^4+^ of GDC lattice were replaced by Mg^2+^ or Fe^2+^ to generate $Mg_{\mathrm{Ce}}^{\prime \prime }$ or $Fe_{\mathrm{Ce}}^{\prime }$ and the positively charged oxygen vacancies ( ${\ddot{V}}_{O}$ ) were generated to maintain the charge balance. The increase in oxygen vacancy concentration of the GDC-M was significantly smaller than that of GDC-F. Similarly, the oxygen vacancy concentration of the GDC-MF was larger than that of GDC-M and slightly smaller than that of GDC-F. This difference was related to the lower solid solubility of MgO ∼1 mol%, resulting in only a small amount of Mg^2+^ entering the CeO_2_ lattice to replace Ce^4+^.

#### 3.1.3 Elemental analysis

**FIGURE 3:**
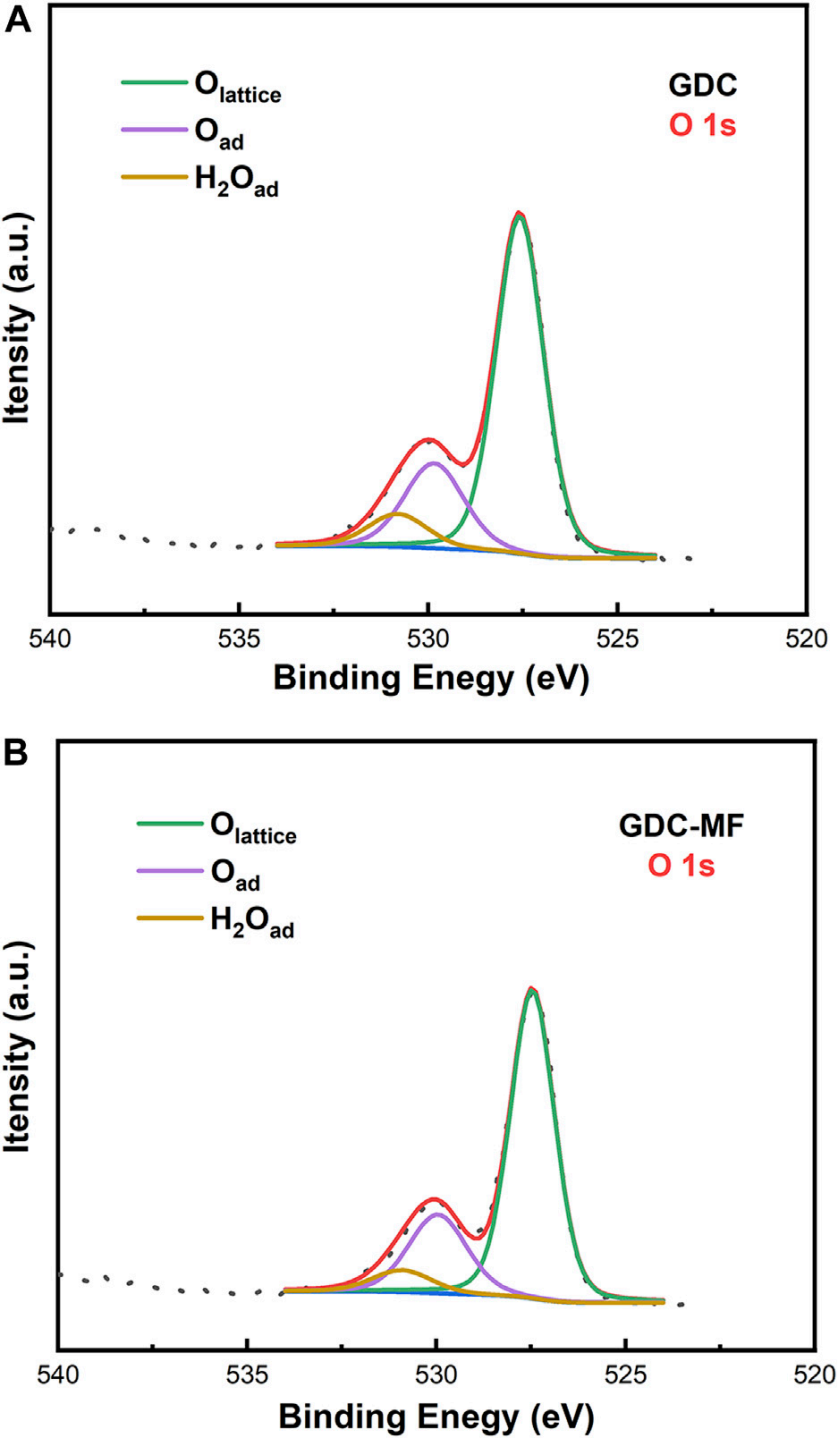
XPS spectra of O 1s for **(A)** GDC and **(B)** GDC-MF samples.

In order to further verify the effect of MgO and Fe_2_O_3_ addition on the oxygen vacancies of GDC, X-ray photoelectron spectroscopy (XPS) was carried out to characterize the chemical environment of the powder surfaces. The fitted XPS spectra of the O 1s orbitals of the GDC and GDC-MF samples were shown in Figure 3. It can be seen that the O 1s spectrum was consisted of three typical peaks located at ∼527.6, ∼530.0 and ∼531.1 eV, representing lattice oxygen (O_lattice_), surface oxygen (O_surface_) (e.g. OH^−^, O^2-^, O^−^ and carbonate) and moist oxygen, respectively (Zhang et al., 2019). A slight increase in the O_surface_/O_lattice_ ratio could be observed, meaning that O_lattice_ was decreased and O_lattice_ vacancy was increased (Bai et al., 2020). According to the fitted peak areas, it can be concluded that the corresponding O_surface_ contents of the GDC and GDC-MF were 23.9 and 25.0%, and the O_surface_ contents were 76.1 and 75.0%, respectively. For the GDC-MF electrolyte, the increase of O_surface_ content was only 1.1%, which was similar to the variation trend estimated by Raman. The reason for its slight increase may be that only small amounts of Mg^2+^ and Fe^3+^ enter the CeO_2_ lattice and Fe_2_O_3_ may connect with some of the oxygen vacancies [ $\mathrm{2F}e_{\mathrm{Ce}}^{\prime }\cdot {\ddot{V}}_{O}$ ], resulting in the consumption of oxygen vacancies (Zheng et al., 2011). With the addition of MgO and Fe_2_O_3_, it can be presumed that MgO/Fe_2_O_3_ mainly played the role of liquid-phase sintering to promote densification. At the same time, only a small portion of Mg^2+^ or Fe^3+^ replaced high-valent Ce^4+^ to produce oxygen vacancies.

#### 3.1.4 Morphology characterization

**FIGURE 4:**
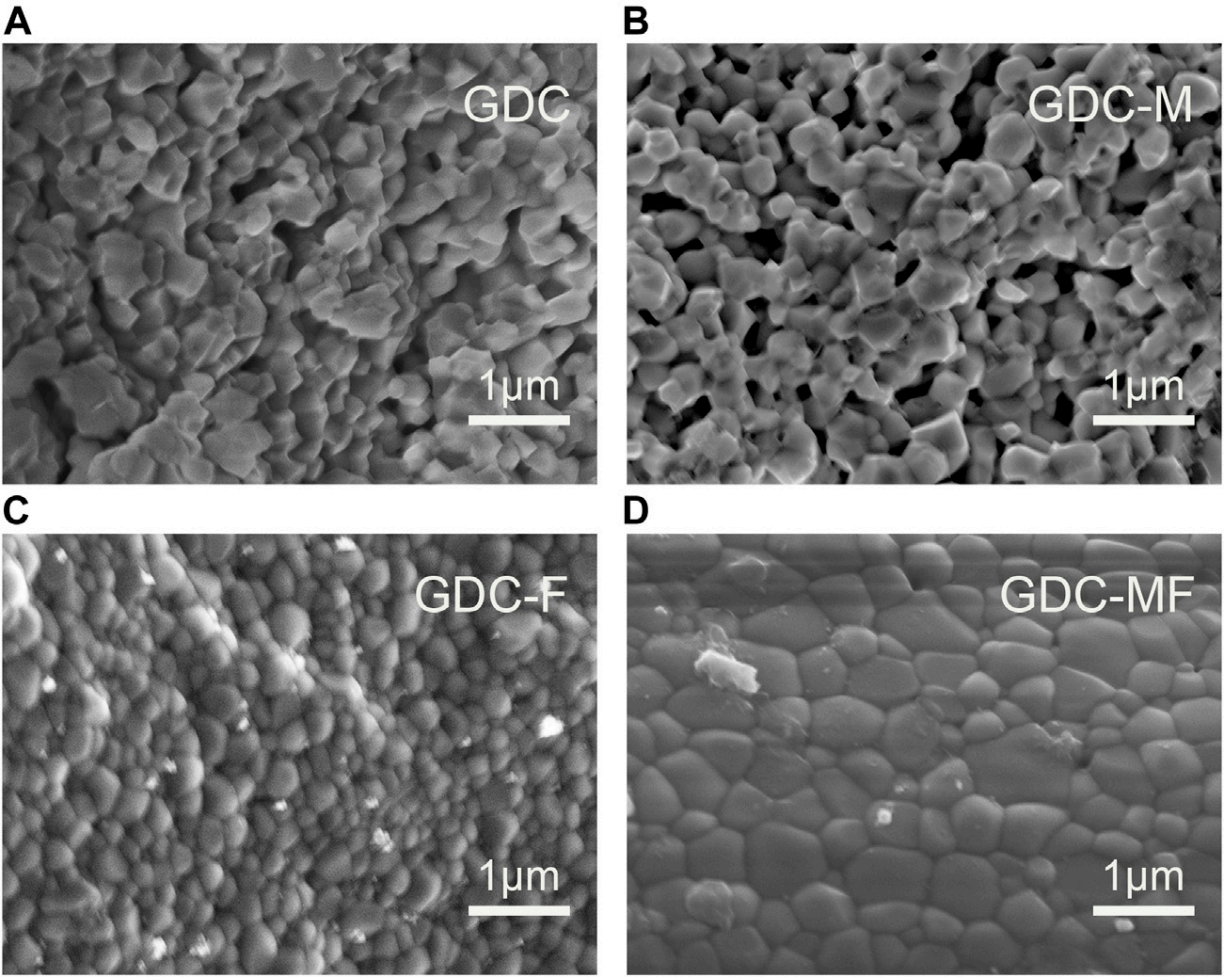
Cross-sectional FE-SEM image of **(A)** GDC, **(B)** GDC-M, **(C)** GDC-F and **(D)** GDC-MF electrolytes sintered at 1200°C for 10 h.

All samples sintered at 1200°C for 10 h were characterized by field emission scanning electron microscopy (FE-SEM). The results of the microscopic morphology of the samples were shown in Figure 4. There were many holes and relatively poor density (86.9%) in the GDC (Figure 4A) samples. The grain sizes of the GDC-M (Figure 4B), GDC-F (Figure 4C) and GDC-MF (Figure 4D) samples were larger than those of GDC (Table 1), indicating that MgO or Fe_2_O_3_ could promote the growth of grains and improve the density of the samples. For the GDC-M samples, the grain size and relative density were larger than those of GDC (Table 1). It is due to the large difference between the ionic radii of Mg^2+^ (0.89 Å) and Ce^4+^ (0.97 Å). During the sintering process, the lattice distortion promoted the movement of grain boundaries, which accelerated the growth of grains and increased the density of the samples. Compared with the GDC-M, the GDC-F had more obvious grain growth and higher relative density (95.1%). On the one hand, this due to the mismatch of the radii of Fe^3+^ (0.78 Å) and Ce^4+^ (0.97Å). On the other hand, it was mainly attributed to the low melting point of Fe_2_O_3_ and the presence of partially melted Fe_2_O_3_ on the grain surface as the sintering temperature increase. Therefore, the surface tension and friction of the grains would be changed, and the rearrangement of the grains would be promoted to make the electrolyte more densified, i.e., the viscous flow mechanism was occurred (Zhang et al., 2004b). The GDC-MF sample had the largest grain size and the highest densities (96.6%), which was produced by the combined action of two sintering aids. Firstly, the densification of the electrolyte was facilitated by the viscous flow mechanism of Fe_2_O_3_. Secondly, due to the difference in the ionic radius of Mg^2+^/Fe^3+^ and Ce^4+^, the GDC lattice distortion and defects were produced, which accelerated the migration of grain boundaries, promoted grain growth, reduced the porosity between grains, and increased the relative density of the electrolyte. Therefore, the co-action of the Fe_2_O_3_ viscous flow mechanism and the radius effect of Mg^2+^/Fe^3+^ and Ce^4+^ were important factors to improve the sintering performance of the GDC-MF electrolyte.

### 3.2 EIS analysis

**FIGURE 5:**
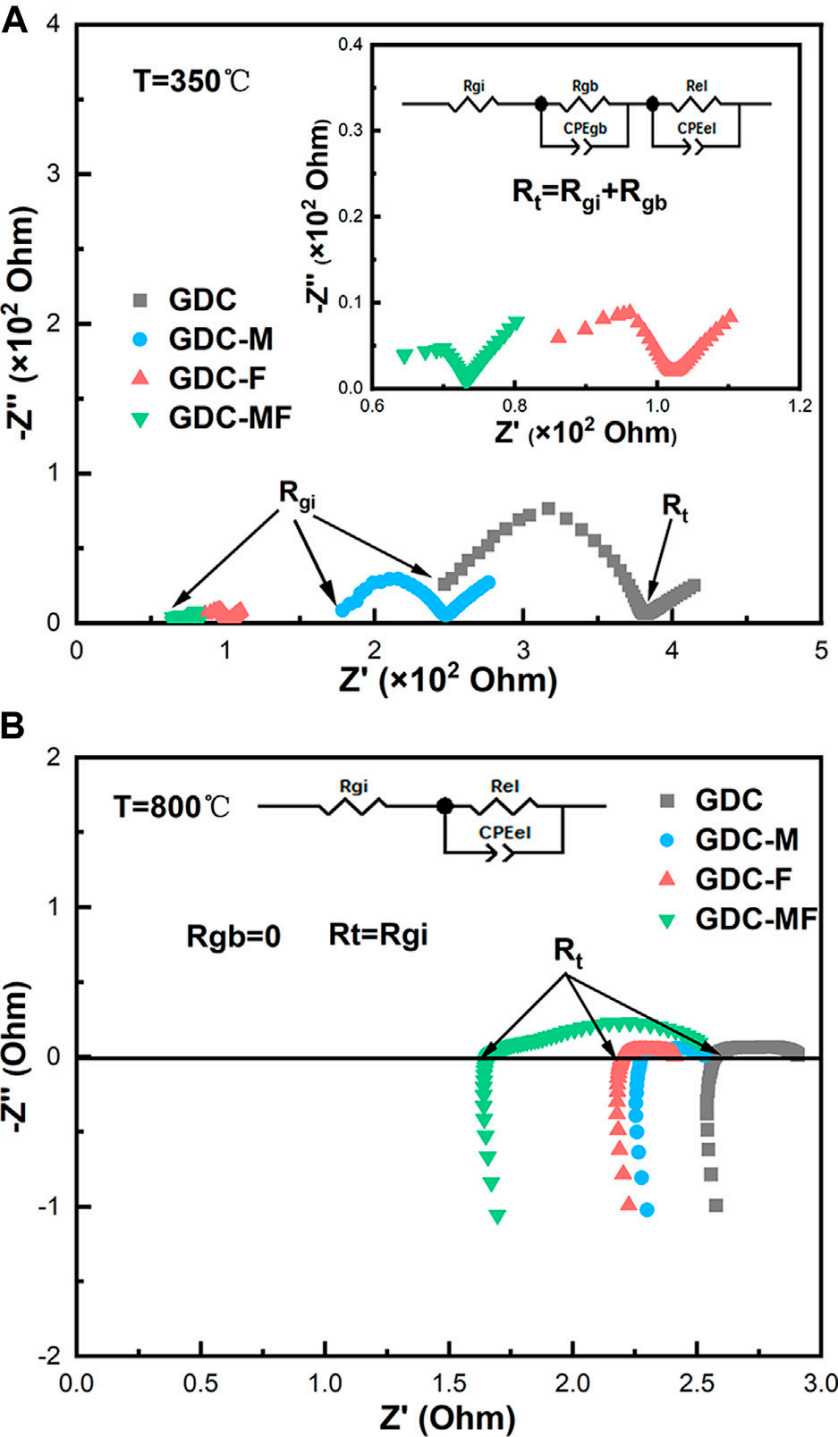
EIS analysis of GDC-based electrolytes and Equivalent circuit diagrams at **(A)** 350 and **(B)** 800°C.

EIS was used to evaluate the electrocatalytic performance of GDC-based electrolytes that were sintered at 1200°C. Figure 5 exhibits the Nyquist spectra and equivalent circuit diagrams of the GDC-based electrolyte measured at 350 and 800°C, where the CPE and R_el_ represent the constant phasor element and polarization resistance, respectively. As shown in Figure 5A, at 350°C, the impedance spectrum was consisted of a semicircle and a small ray arc. The plots could be divided into high and low frequency arcs, and the intersection of the high frequency arc and the low frequency arc with the real axis (Z′) were the grain resistance (R_gi_) and the total resistance (R_t_). The difference between the R_t_ and the R_gi_ was the grain boundary resistance (R_gb_). As the temperature increased to 800°C (Figure 5B), the impedance spectrum was changed to a ray arc with a tail, and the R_gb_ was disappeared, where R_t_ = R_gi_. The associated resistance data were listed in Table 2. Combining Figure 5A and Table 2, it can be seen that the R_gi_, R_gb_ and R_t_ of the GDC-M, GDC-F and GDC-MF were lower than that of GDC electrolyte (R_GDC-MF_ < R_GDC-F_ < R_GDC-M_ < R_GDC_). At 350°C, the R_gb_ (3.14 Ω) and R_gi_ (70.03 Ω) of the GDC-MF were only 4.85 and 22.11% of the GDC electrolyte, indicating that the co-addition of MgO and Fe_2_O_3_ could effectively reduce the R_gb_ and R_gi_. Comparing the R_gi_/R_t_ of the GDC-M, GDC-F and GDC-MF, it can be seen that the ratio of R_gb_ to R_t_ of the GDC-MF (0.04) was significantly lower than that of GDC-M (0.15) and GDC-F (0.06). This indicated that the addition of Fe_2_O_3_-MgO dual sintering aids in GDC electrolyte was exhibited a more significant effect on reducing the R_gb_. This was attributed to the liquid phase sintering mechanism of Fe_2_O_3_ (Wang et al., 2017), which increased the effective densification of the sample and reduced the R_gi_ and R_gb_, thereby reducing the overall resistance. In addition, the radius effect of Mg^2+^ and Fe^3+^ was also an important factor to improve the sintering performance and electrical conductivity of GDC-MF.

**TABLE 2:** Resistance of GDC-based electrolytes at 350 and 800°C.

Samples	350°C				800°C
	*R*_gi_ (Ω)	*R*_gb_ (Ω)	*R*_t_ (Ω)	*R*_gb_/*R*_t_	*R*_t_ = *R*_gi_(Ω)
GDC	316.79	64.79	381.58	0.17	2.57
GDC-M	215.99	32.04	248.03	0.13	2.28
GDC-F	96.16	6.05	102.21	0.06	2.20
GDC-MF	70.03	3.14	73.174	0.04	1.66

### 3.3 Conductivity analysis

**FIGURE 6:**
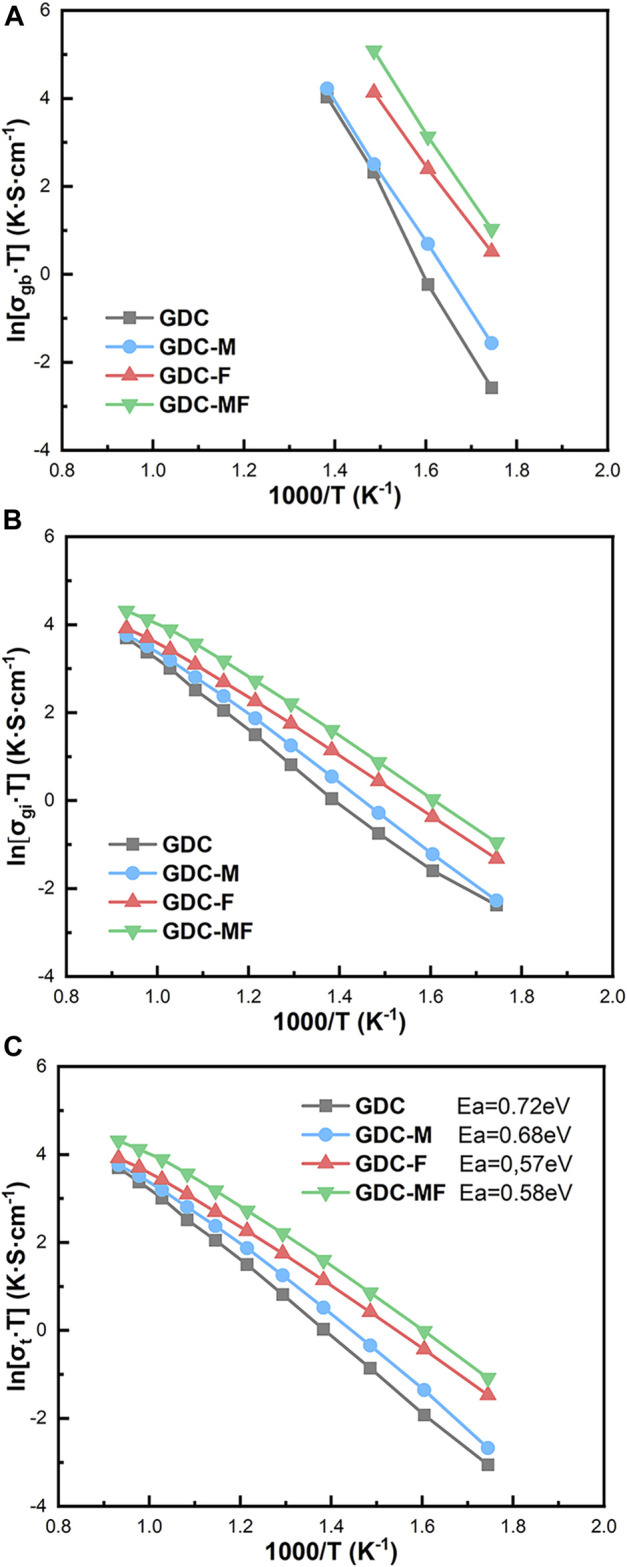
Arrhenius curves of **(A)** σ_gb_, **(B)** σ_gi_, and **(C)** σ_t_ of GDC-based electrolytes.

Figures 6A–C was the Arrhenius curve of the grain boundary conductivity (σ_gb_), grain conductivity (σ_gi_), and total conductivity (σ_t_) of GDC-base electrolytes. It can be seen from Figure 6 that in the temperature range of 300–800°C, the conductivity of the GDC electrolyte was always the lowest. Compared with the GDC electrolyte, the σ_gi_ and σ_gb_ of the GDC-M, GDC-F and GDC-MF were improved significantly, and the GDC-MF had the maximum σ_gi_, σ_gb_ and σ_t_. At 400°C, the σ_gi_ and σ_gb_ of the GDC-MF were 3.56 × 10^−3^ S cm^−1^ and 2.40 × 10^−1^ S cm^−1^, which were 5.42 and 15.89 times higher than the σ_gi_ (6.57 × 10^−4^ S cm^−1^) and σ_gb_ (1.51 × 10^−2^ S cm^−1^) of the GDC. Additionally, the increase of σ_gb_ was more significant. At 800°C, the conductivity of the GDC-MF (6.97 × 10^−2^ S cm^−1^) was much higher than the conductivity of 2.5 mol% CoO-Bi_2_O_3_ co-doped NDC (5.765 × 10^−2^ S cm^−1^) (Chen et al., 2020) and the Ce_0.8_Gd_0.2-X_Sr_x_O_1.9-x/2_ (x = 0.01) composite electrolyte (3.6 × 10^–2^ S cm^−1^) (Kashyap et al., 2014). And the σ_t_ of GDC electrolyte with over 95% relative density calcined at 1400°C was 3.25 × 10^−2^ S cm^−1^, which was only 46% of the GDC-MF electrolyte (Öksüzömer et al., 2013). Supplementary Table S1 summarize the total conductivity comparison of different electrolytes. In addition, the total conductivities of GDC and GDC-MF under different partial oxygen pressures were also explored (Supplementary Figure S1), as discussed in detail in Supplementary Material. The corresponding activation energy (E_a_) was calculated according to the Arrhenius formula and expressed in Figure 6C. From the picture, the GDC-MF (E_a_ = 0.58 eV) was showed the lowest activation energy similar to the GDC-F (E_a_ = 0.57 eV), but lower than the GDC (E_a_ = 0.72 eV) and GDC-M (E_a_ = 0.68 eV). The reduction in activation energy was meant that high ionic conductivity could be achieved at reduced temperatures. In summary, the improvement of GDC based-electrolytes performance could be attributed to: (I) The liquid phase sintering mechanism of MgO/Fe_2_O_3_ promoted the movement of grain boundaries and improved the densification of the samples, thus reducing the grain/R_gb_; (II) The molten Fe_2_O_3_ had good continuity and permeability at the GDC grain boundary, which could effectively promote the migration of oxygen ions; (III) The MgO remaining at the grain boundary could remove the SiO_2_ impurities (Bi et al., 2017), change the structure of the space charge layer (Zhang et al., 2004b), and improve the conductivity of the grain boundary; (IV) A small amount of Mg^2+^ or Fe^2+^ could enter the lattice to generate oxygen vacancies, provide oxygen ion transport channels and improve the σ_gi_.

### 3.4 Sintering mechanism

**FIGURE 7:**
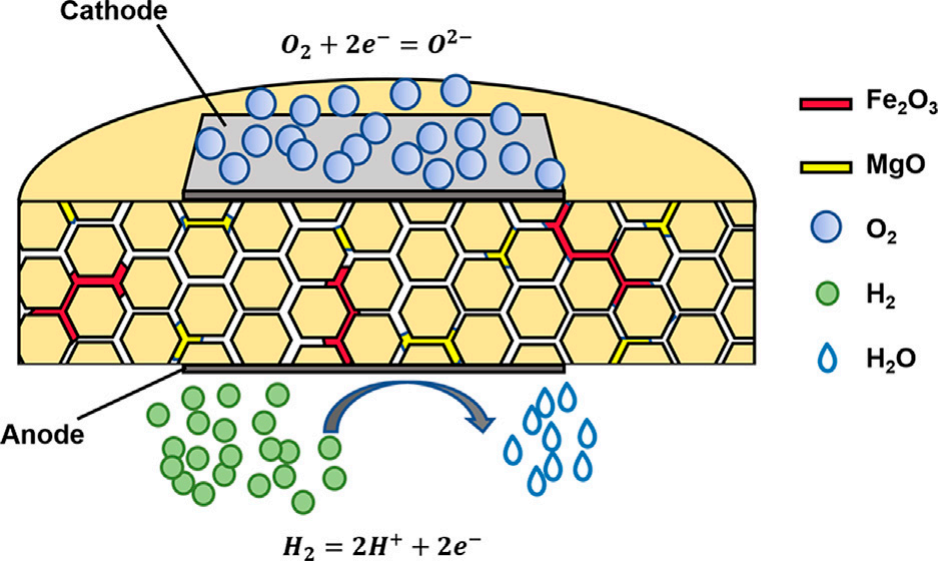
Schematic diagram of the full-cell model supported by GDC-MF electrolyte.

To illustrate the excellent performance of GDC-MF electrolyte, the possible mechanism for the improvement of conductive was proposed in Figure 7. The Fe_2_O_3_ and MgO sintering aids were represented by red and golden yellow skeleton, respectively. During the sintering process, the Fe_2_O_3_/MgO particles were gradually melted at the grain boundaries to form a liquid phase, which promoted the sintering process by diffusing and surrounding the GDC particles. After sintering, the cross-section of GDC-MF was analyzed by EDX mapping (Supplementary Figure S2), and it was found that the distribution of Ce, Gd and Fe elements contained in the sample was relatively uniform, indicating that Fe^3+^ easily entered the GDC lattice. In addition, line scan analysis (Supplementary Figure S3) showed that the content of Mg element in the grain boundaries was higher than that in the grains. This is due to the low solid solubility of MgO ∼1 mol%, and only a small amount of Mg^2+^ enters the CeO_2_ lattice (Cho et al., 2007). Other MgO might act as a grain boundary scavenger and interact with SiO_2_ to generate Mg_2_SiO_4_, reducing the grain boundary resistance, thereby increasing the grain boundary conductivity of GDC (Bi et al., 2017). However, there is no peak of Mg_2_SiO_4_ in XRD due to the relatively small amount. Therefore, the addition of Fe_2_O_3_/MgO dual sintering aids in GDC electrolyte proposed in this work was an effective strategy to obtain highly dense electrolyte materials.

### 3.5 Physical and chemical properties

Previously, the GDC-MF composite electrolytes with high ionic conductivity and good densification have been demonstrated. In addition, the GDC-MF electrolytes should have good TEC, chemical compatibility, oxygen reduction reaction (ORR) activity and long-term stability (Zhang et al., 2004c; Zhu et al., 2017).

#### 3.5.1 Thermal expansion coefficient

**FIGURE 8:**
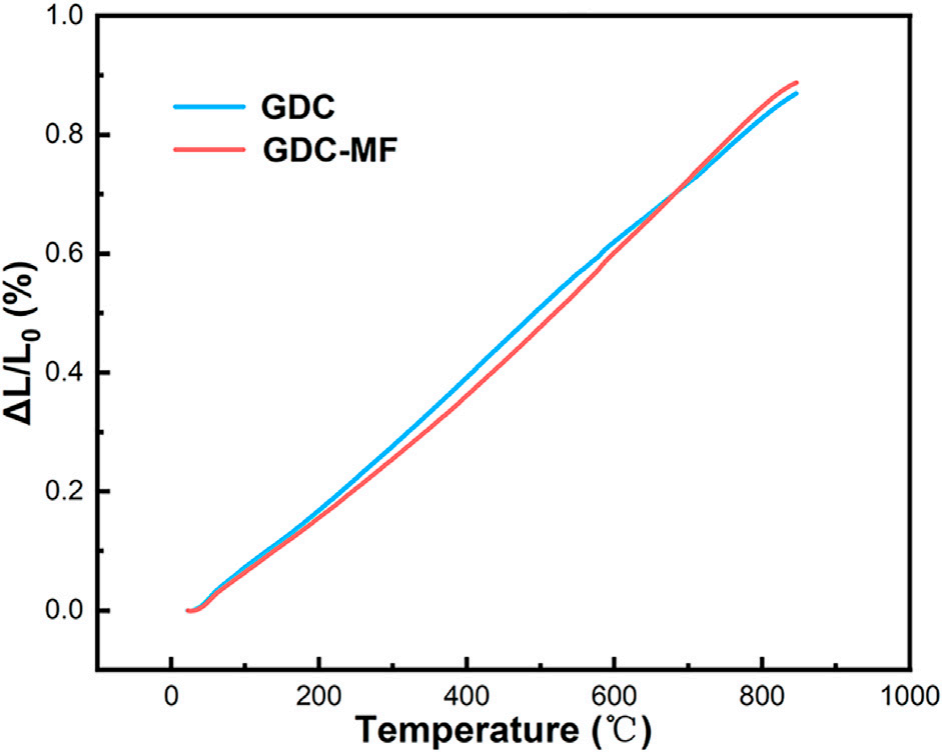
Thermal expansion curves of sintered samples versus temperature.

The thermal expansion behavior of GDC and GDC-MF electrolytes in the temperature range of 50–800°C was shown in Figure 8. The average TEC was calculated by fitting the dL/L_0_ data as the function of temperature. As shown in Figure 8, the thermal expansion curve of the electrolyte was increased with the increase of temperature, and the average TEC of GDC and GDC-MF was calculated to be 10.9 × 10^−6^ K^−1^ and 11.2 × 10^−6^ K^−1^, respectively. The GDC and GDC-MF electrolytes were exhibited similar TECs with LSCF (15.4 × 10^−6^ K^−1^) cathode (Tai et al., 1995) and NiO-based anode (16.9 × 10^−6^ K^−1^) (Chen et al., 2008) materials. The results indicate that the addition of Fe_2_O_3_-MgO dual sintering aids had no effect on the TEC of the GDC electrolyte. The GDC-MF and GDC electrolytes have good thermal matching with the electrodes, and each component will not break in the SOFC operating temperature range.

#### 3.5.2 Chemical compatibility

**FIGURE 9:**
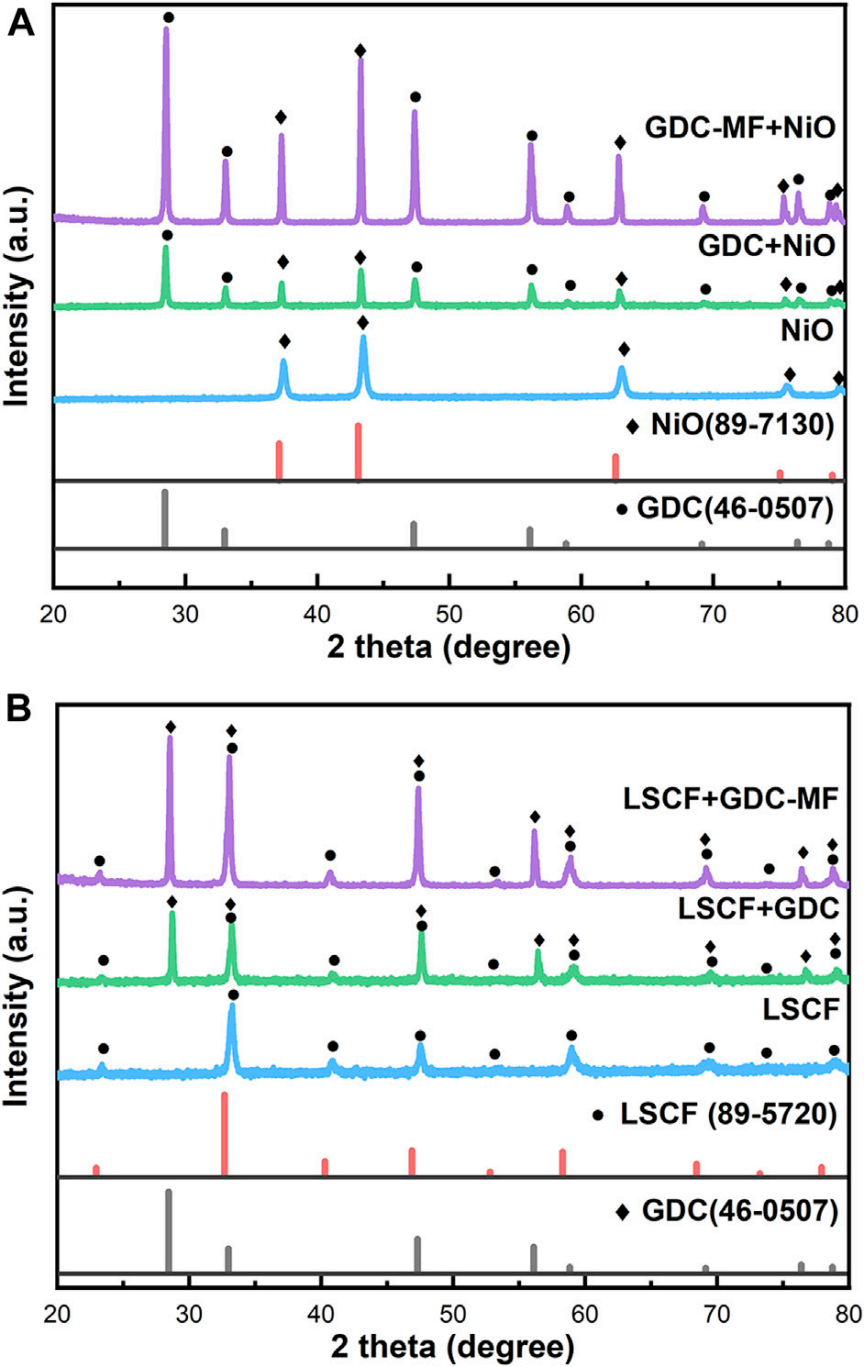
Chemical compatibility of the GDC-MF electrolyte with **(A)** LSCF cathode and **(B)** NiO anode material.

The chemical compatibility between the perovskite cathode material LSCF and NiO anode with the GDC and GDC-MF electrolyte was studied. The GDC and GDC-MF electrolyte powders were mixed with NiO anode powder and LSCF cathode powder at the weight ratio of 1:1, respectively, and calcined at 950°C for 15 h in air. The XRD patterns measured at room temperature were shown in Figures 9A,B. It can be seen that the main diffraction peaks could be assigned to the characteristic diffraction peaks of GDC, GDC-MF, NiO and LSCF, and no new diffraction peaks were generated. It was confirmed that the GDC-MF electrolyte exhibits good chemical compatibility with NiO and LSCF electrodes at the high temperature of 950°C.

#### 3.5.3 Oxygen reduction activity

**FIGURE 10:**
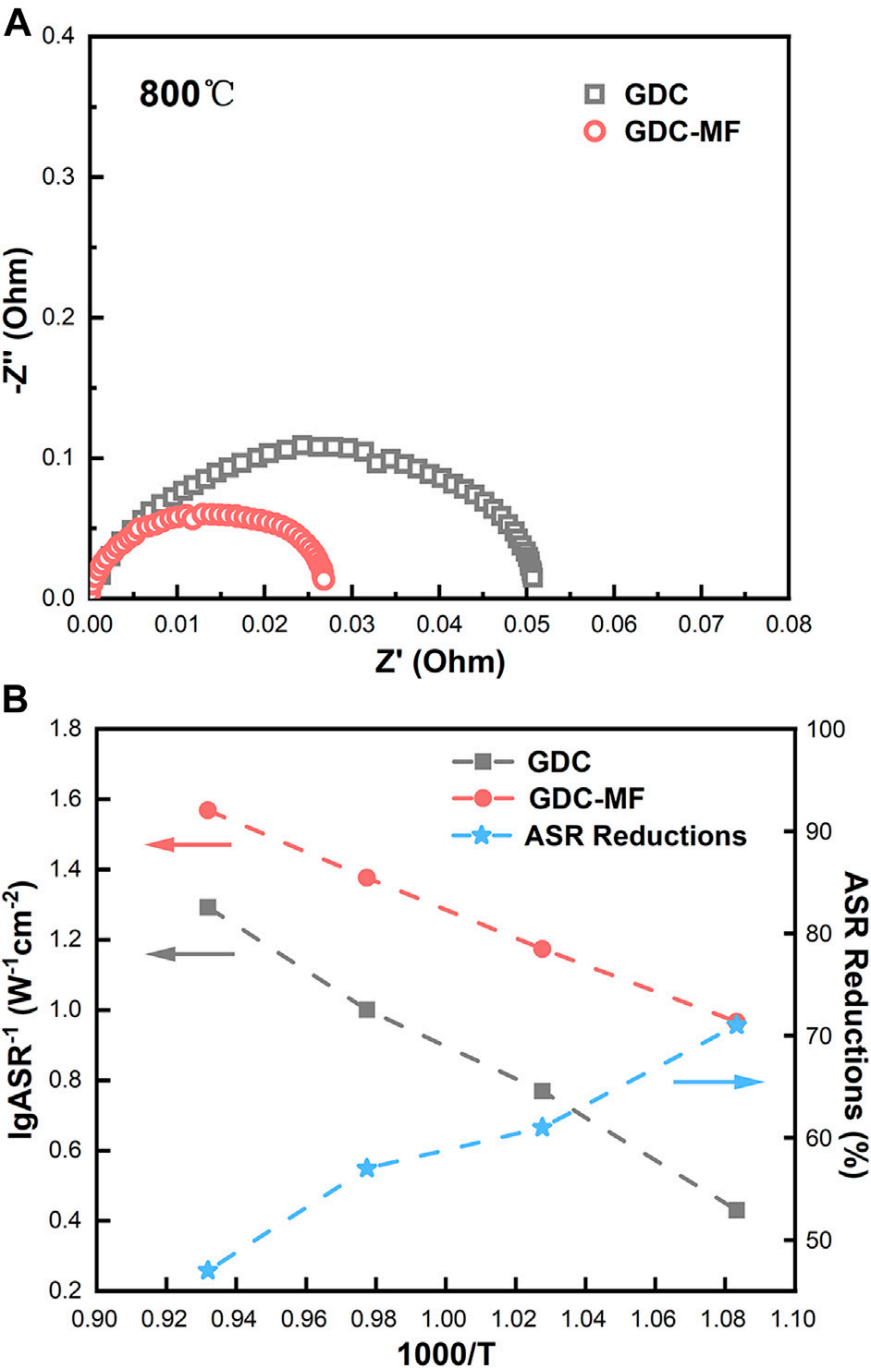
Electrochemical performance of symmetric cells: **(A)** Electrochemical impedance spectra at 800 °C. **(B)** ASR versus temperature curves of LSCF cathodes on GDC and LSCF cathodes on GDC-MF (left *Y*-axis), and percentage reduction of ASR of LSCF cathodes on GDC-MF relative to GDC (right *Y*-axis).

To evaluate the effect of the composite electrolyte on the ORR activity at the cathode/electrolyte interface, electrochemical impedance spectroscopy tests of symmetrical cells (LSCF/GDC/LSCF and LSCF/GDC-MF/LSCF) were performed in the temperature range of 650–800°C. As shown in Figure 10A, the ohmic area-specific resistance (ASR_oh_) corresponds to the real axis intercept in the high frequency region, and the total area-specific resistance (ASR_t_) corresponds to the real axis intercept in the low frequency region. The polarization area-specific resistance (ASR_p_) can be estimated according to the formula $\mathrm{AS}R_{t}\mathrm{-AS}R_{\mathrm{oh}}\mathrm{=AS}R_{P}$ .

The ASR_p_ value versus temperature is shown in Figure 10B. It can be seen that the ASR_p_ of the symmetric cells assembled with GDC-MF electrolytes was significantly decreased in the temperature range of 650–800 °C compared to the GDC electrolyte. Furthermore, the ASR_p_ value of LSCF in GDC-MF electrolytes was reduced by more than 47% compared to GDC (right *y*-axis). It is shown that adding Fe_2_O_3_-MgO dual sintering aids effectively reduced the ASR_p_ between the GDC-based electrolyte and the LSCF cathode and increased the ORR rate. The enhanced ORR rate could be attributed to the improved density of the GDC electrolyte with the addition of Fe_2_O_3_ and MgO.

#### 3.5.4 Single cell electrochemical performance

**FIGURE 11:**
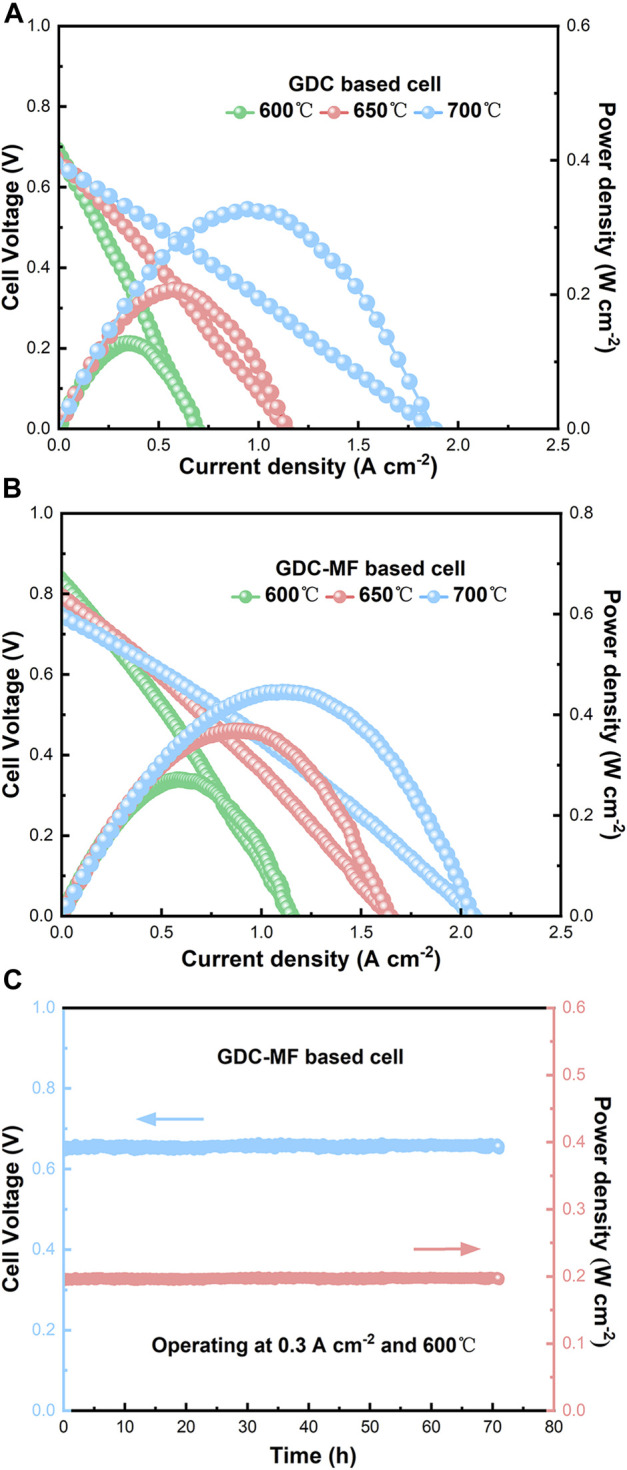
Electrochemical performance measured at 600°C, 650°C, and 700°C of **(A)** the GDC, **(B)** GDC-MF electrolyte-based SOFC, and **(C)** long-term stability of Cell-II measured at 600°C for 70 h.

The electrolyte-supported single cells were assembled to evaluate the effect of Fe_2_O_3_ and MgO on the output performance of GDC-based SOFCs. NiO and LSCF powders were used as anode and cathode materials. The single cells supported with GDC and GDC-MF electrolytes were labelled as Cell-I and Cell-II, respectively. It was compared the current density-voltage (I-V) and current density-power density (I-P) curves of single cells Cell-I and Cell-II in the temperature range of 600–700°C, using wet H_2_ as the fuel and air as the oxidant (Figures 11A,B). The maximum OCV of Cell-I and Cell-II was 0.71 and 0.83 V, respectively. It can be seen that the maximum OCVs of the 2 cells were lower than the theoretical OCV of 1.04–1.1 V (Steele, 2000; Leah et al., 2005). This is mainly attributed to that part of Ce^4+^ in the electrolyte was reduced to Ce^3+^ at low oxygen partial pressure (under reducing atmosphere) and high temperature, resulting in a certain n-type electronic conductivity. Due to the existence of electrons, a short-circuit current is formed inside the electrolyte, The short-circuit current was formed inside the electrolytes due to the presence of electrons, so that its OCVs were less than the theoretical electromotive force (Zhou et al., 2022). In addition, the OCV of the single cells decreased slightly with increasing temperature, which was conformed to the trend predicted by the Nernst equation (Zhao et al., 2014). The peak power densities of Cell-I at 600°C, 650 and 700°C were 0.13, 0.21, and 0.33 W cm^−2^, and the peak power densities of Cell-II were 0.27, 0.37 and 0.45 W cm^−2^, respectively. Compared with Cell-Ⅰ, the peak power densities of cell-Ⅱ have been greatly improved. At 700°C, the peak power density of Cell-II was 36.4% higher than that of Cell-Ⅰ. The improved power density of Cell-II could be attributed to the enhanced electrolyte density, improved electrical conductivity, matching of TEC, and excellent ORR rate. As shown in Figure 11C, Cell-II was tested for long-term stability at 600°C and the current density of 0.3 A cm^−2^ for 70 h. It can be seen from the figure that the voltage and power density of the single cell did not change during the test time. These results confirm that the GDC-MF electrolyte is a promising electrolyte material for IT-SOFC.

## 4 Conclusion

In summary, the effects of the addition of MgO and Fe_2_O_3_ on the sintering behavior, phase composition, microstructure, electrochemical performance and compatibility with classical electrodes of GDC electrolytes were systematically investigated. All the GDC-based electrolytes samples were prepared by a sol-gel method and exhibited the cubic fluorite structure. MgO-Fe_2_O_3_ acted as a dual sintering aid could increase the effective densification of the samples and reduce the R_gi_, R_gb_ and R_t_ of the GDC-MF electrolyte. At 400 °C, the σ_gb_ and σ_t_ of the GDC-MF were 2.40 × 10^−1^ S cm^−1^ and 3.50 × 10^−3^ S cm^−1^, which were 15.89 and 5.56 times higher than those of GDC σ_gb_ (1.51 × 10^−2^ S cm^−1^) and σ_t_ (6.30 × 10^−4^ S cm^−1^). Additionally, the GDC-MF electrolyte had matchable thermal expansion coefficient and excellent chemical compatibility with commonly used electrodes. Furthermore, the single cell supported by GDC-MF electrolyte could reach the highest power density of 0.45 W cm^−2^ at 700°C, and demonstrate the stable voltage and power density in the long-term stability test within 70 h. Therefore, adding Fe_2_O_3_-MgO dual sintering aids to GDC electrolyte is an effective approach to improve the electrochemical performance, and the GDC-MF electrolyte should be a promising electrolyte for IT-SOFC.

## Data Availability

The original contributions presented in the study are included in the article/Supplementary Material, further inquiries can be directed to the corresponding authors.

## Glossary

ASR_p_: Polarization area-specific resistance
Cell-I: Single cells supported with GDC electrolyte
Cell-II: Single cells supported with GDC-MF electrolyte
E_a_: Activation energy
EIS: Electrochemical impedance spectroscopy
FE-SEM: Field emission scanning electron microscopy
GDC: Gd_0.2_Ce_0.8_O_2–δ_
GDC-F GDC: Samples with 4 mol% Fe_2_O_3_ addition
GDC-M GDC: Samples with 4 mol% MgO addition
GDC-MF GDC: Samples with 2 mol% MgO+2 mol% Fe_2_O_3_ addition
IT-SOFCs: Intermediate-temperature solid oxide fuel cells
LSCF: La_0.6_Sr_0.4_Co_0.8_Fe_0.2_O_3-δ_
OCV: Open-circuit voltage
O_lattice_: Lattice oxygen
ORR: Oxygen reduction reaction
O_surface_: Surface oxygen
R_gb_: Grain boundary resistance
R_gi_: Grain resistance
R_t_: Total resistance
SOFCs: Solid oxide fuel cells
TEC: Thermal expansion coefficient
XPS: X-ray photoelectron spectroscopy
XRD: X-ray diffractometer
σ_gb_: Grain boundary conductivity
σ_gi_: Grain conductivity
σ_t_: Total conductivity
