# Supplementary figures and images for: Effect of MgO and Fe2O3 dual sintering aids on the microstructure and electrochemical performance of the solid state Gd0.2Ce0.8O2-δ electrolyte in intermediate-temperature solid oxide fuel cells
Source: Front Chem. 2022 Sep 27;10:991922. doi: 10.3389/fchem.2022.991922 (PMC9550866; doi:10.3389/fchem.2022.991922)

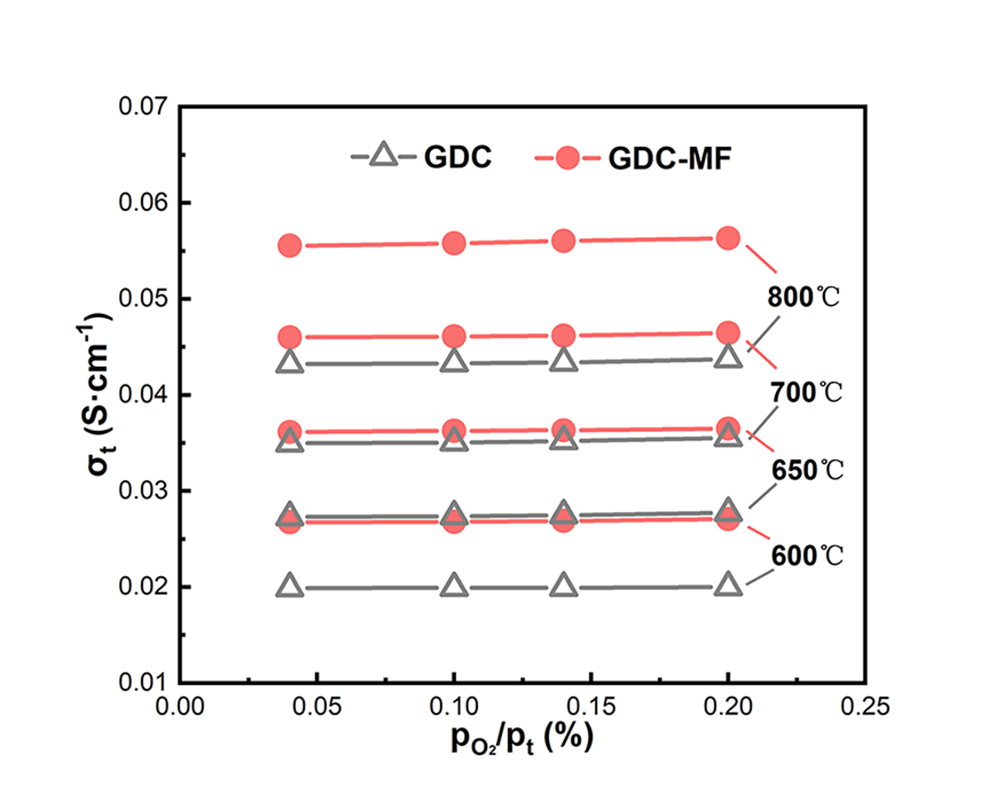

Supplement: Supplementary file 1 [file DataSheet2.zip › Figure S1.tif]

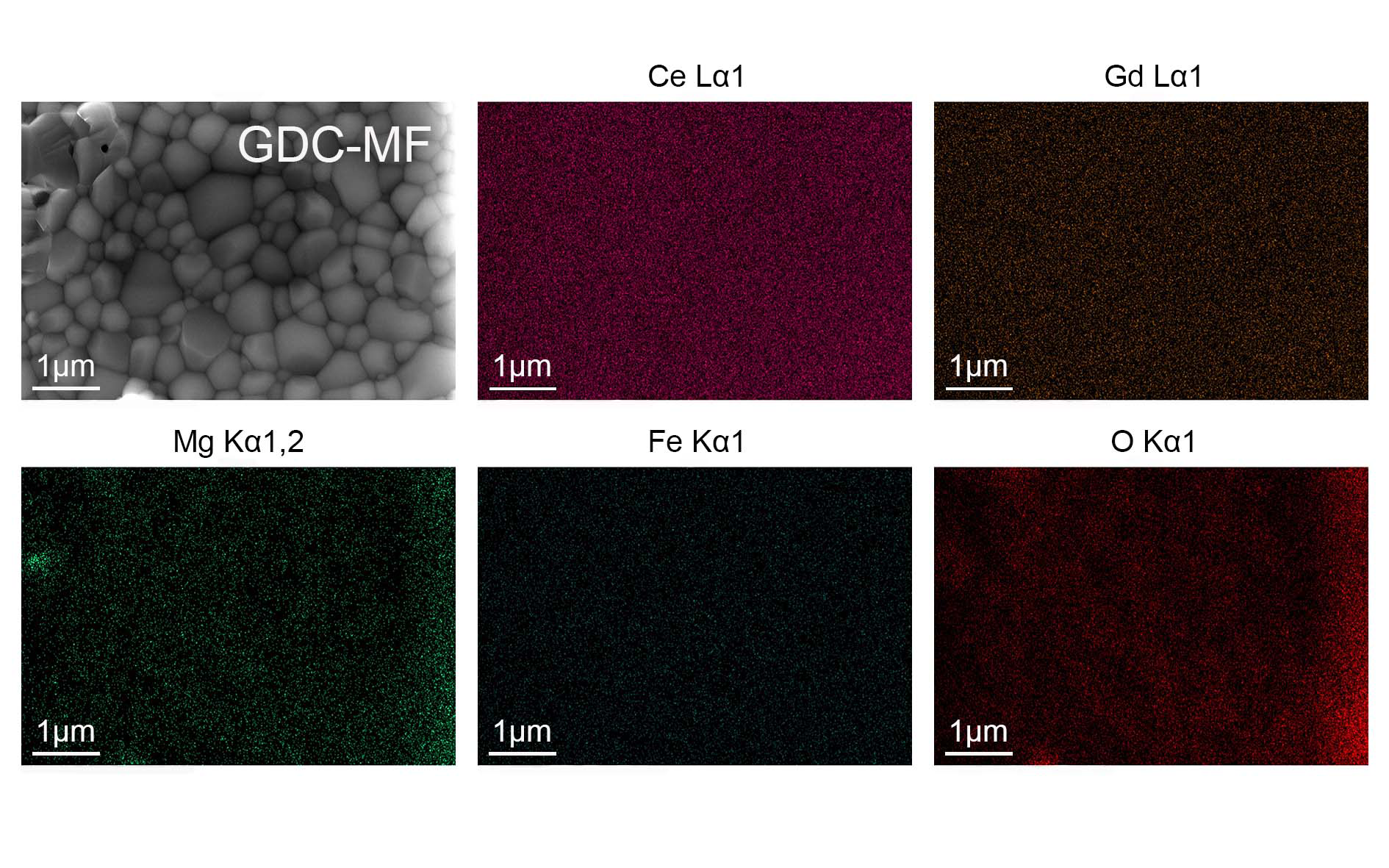

Supplement: Supplementary file 1 [file DataSheet2.zip › Figure S2.tif]

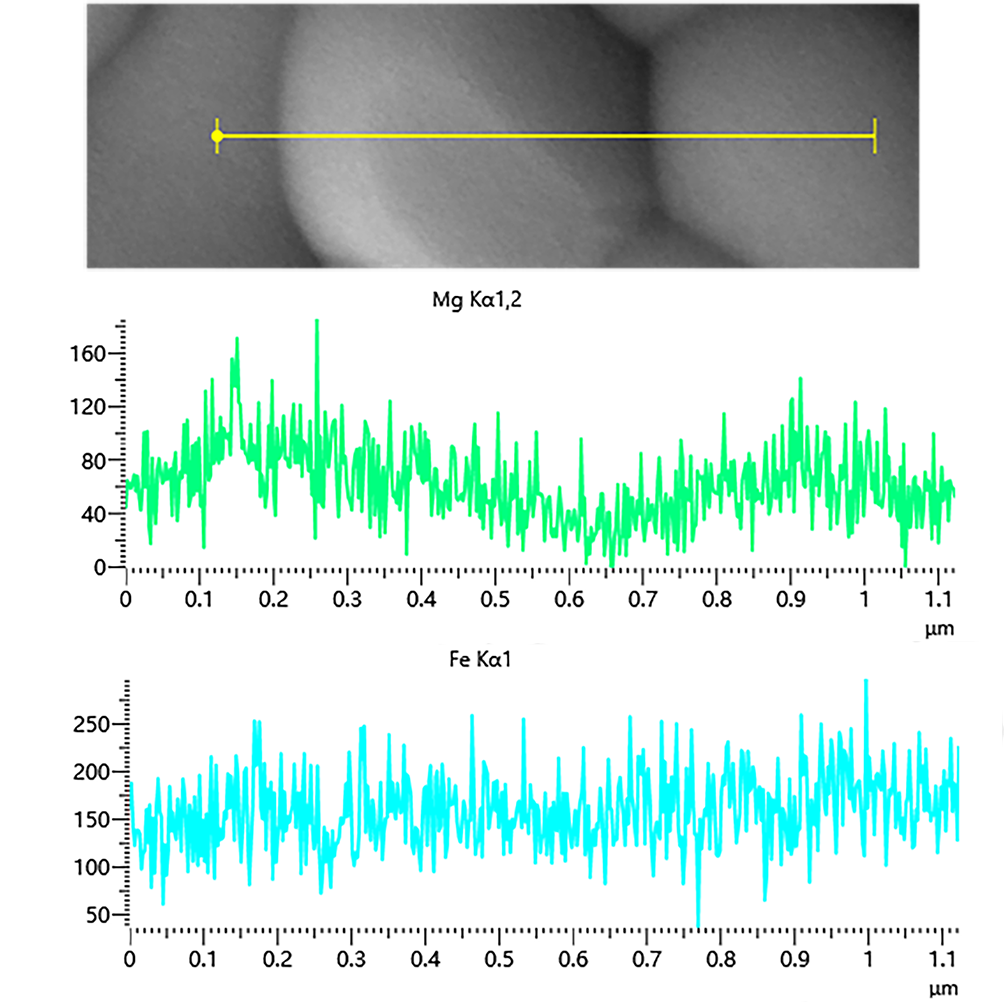

Supplement: Supplementary file 1 [file DataSheet2.zip › Figure S3.tif]
