# Supplementary material for: Effect of MgO and Fe2O3 dual sintering aids on the microstructure and electrochemical performance of the solid state Gd0.2Ce0.8O2-δ electrolyte in intermediate-temperature solid oxide fuel cells
Source: Front Chem. 2022 Sep 27;10:991922. doi: 10.3389/fchem.2022.991922 (PMC9550866; doi:10.3389/fchem.2022.991922)
Supplement: Supplementary file 2 [file DataSheet1.docx]

Supplementary Material

# Conductivity Analysis

The total conductivities of GDC and GDC-MF electrolytes were obtained at different oxygen partial pressures and different temperatures, as shown in Figure S1. As can be seen in the Figure, the total conductivity of GDC-MF is greater than the total conductivity of the GDC electrolyte at any one of the same temperatures and the same oxygen partial pressure. And with the increase of temperature, the difference between the total conductivity of GDC-MF and GDC electrolyte under the same oxygen partial pressure was gradually larger.

To further distinguish the contribution of different charge carriers to the conductivity of the GDC-MF electrolyte (Wang et al., 2021). The electronic conductivity (σ_e_) and ionic conductivity (σ_i_) of the GDC-MF electrolyte were analyzed. In the GDC electrolytes, the electron transport path through lattice oxygen was shown in Eq. (1):

$\boldsymbol{Ce}^{\mathbf{4}+}-\boldsymbol{O}-\boldsymbol{Ce}^{\mathbf{3}+}$ (1)

However, after adding MgO and Fe_2_O_3_ to CeO_2_, there would be a small amount of Mg^2+^ or Fe^3+^ to replace Ce^4+^, resulting in the replacement of lattice oxygen by oxygen vacancies, which cut off the transmission path of electrons. Therefore, the electronic conductivity of the GDC-MF electrolyte was decreased. Oxygen vacancies ($\boldsymbol{V}_{\boldsymbol{O}}^{\boldsymbol{\cdot\cdot}}$) had two positive charges and were easily attracted to O^2-^, which increased the transport pathway of the O^2-^ and increased the ionic conductivity of the GDC-MF electrolyte. Furthermore, the ionic conductivity could be determined according to $\boldsymbol{\sigma}_{\boldsymbol{i}}\mathbf{=}\boldsymbol{\sigma}_{\boldsymbol{t}}\boldsymbol{-}\boldsymbol{\sigma}_{\boldsymbol{e}}$. The overall conductivity of the GDC-MF was increased while the electronic conductivity was decreased, which could be concluded that the ionic conductivity of the GDC-MF was increased. Therefore, the GDC-MF electrolyte exhibited good ionic conductivity.

# Reference

Accardo, G., Frattini, D., Ham, H.C., Han, J.H., and Yoon, S.P. (2018). Improved microstructure and sintering temperature of bismuth nano-doped GDC powders synthesized by direct sol-gel combustion. *Ceram. Int.* 44 3800–3809.

Anjaneya, K.C., and Singh, M.P. (2017). Synthesis and properties of gadolinium doped ceria electrolyte for IT-SOFCs by EDTA-citrate complexing method. *Journal of Alloys and Compounds* 695**,** 871-876.

Babar, Z.U.D., Hanif, M.B., Gao, J.-T., Li, C.-J., and Li, C.-X. (2022). Sintering behavior of BaCe_0.7_Zr_0.1_Y_0.2_O_3-δ_ electrolyte at 1150 °C with the utilization of CuO and Bi_2_O_3_ as sintering aids and its electrical performance. *International Journal of Hydrogen Energy* 47**,** 7403-7414.

Ding, H., Qu, D., Sun, H., Guo, X., Li, J., Li, Q., Li, G., Wang, P., and Zhang, X. (2019). Improved sintering behavior and electrical performance of Ce_0.8_Sm_0.2_O_2-δ_ - BaZr_0.1_Ce_0.7_Y_0.2_O_3-δ_ (SDC -BZCY) composite electrolytes with the addition of iron (III) oxide for IT-SOFCs. *Ceramics International* 45**,** 24702-24706.

Li, Y., Yang, W., Wang, L., Zhu, J., Meng, W., He, Z., and Dai, L. (2018). Improvement of sinterability of BaZr_0.8_Y_0.2_O_3-δ_ for H_2_ separation using Li_2_O/ZnO dual-sintering aid. *Ceramics International* 44**,** 15935-15943.

Lima, C.G.M., Santos, T.H., Grilo, J.P.F., Dutra, R.P.S., Nascimento, R.M., Rajesh, S., Fonseca, F.C., and Macedo, D.A. (2015). Synthesis and properties of CuO-doped Ce_0.9_Gd_0.1_O_2−δ_ electrolytes for SOFCs. *Ceramics International* 41**,** 4161-4168.

Wang, H., Zhang, W., Meng, J., Pei, Y., Qiu, X., Meng, F., and Liu, X. (2021). Effectively Promoting Activity and Stability of a MnCo_2_O_4_‑Based Cathode by In Situ Constructed Heterointerfaces for Solid Oxide Fuel Cells. *ACS Appl. Mater. Interfaces* 13**,** 24329−24340.

Wang, J., Chen, X., Xie, S., Chen, L., Wang, Y., Meng, J., and Zhou, D. (2019). Bismuth tungstate/neodymium-doped ceria composite electrolyte for intermediate-temperature solid oxide fuel cell: Sintering aid and composite effect. *Journal of Power Sources* 428**,** 105-114.

Xia, Y., Bai, Y., Wu, X., Zhou, D., Wang, Z., Liu, X., and Meng, J. (2012). Effect of sintering aids on the electrical properties of Ce_0.9_Nd_0.1_O_2−δ_. *Solid State Sciences* 14**,** 805-808.

Zhou, J., Tang, P., Bai, J.-H., Chen, Y.-X., Meng, Y., Zhu, X.-F., Wang, N., Zhou, D.-F., and Yan, W. (2022). A new type of Gd_0.2_Ce_0.8_O_3-δ_ fuel cell electrolyte containing Er_0.2_Bi_0.8_O_1.5_ with highly improved performance. *Journal of Alloys and Compounds* 901.

# Supplementary Figures and Tables

## Supplementary Figures

###### Supplementary Figure S1. Variation of total conductivity of the GDC and GDC-MF electrolytes at different temperatures and different oxygen partial pressures.

###### Supplementary Figure S2. EDX mapping image of GDC-MF electrolyte.

###### Supplementary Figure S3. EDX diagram of line scan.

## Supplementary Tables

###### Supplementary Table S1. The total conductivity comparison of different electrolytes.

| Electrolyte | Sintering temperature | σ_t_ (S/cm) | | Ref |
| --- | --- | --- | --- | --- |
|  |  | 600 ℃ | 700 ℃ |  |
| GDC | 1200 ℃ | 8.89×10^-3^ | 2.08×10^-2^ | This work |
| GDC-MF | 1200 ℃ | 2.75×10^-2^ | 5.03×10^-2^ | This work |
| Ce_0.9_Gd_0.1_O_2-δ_(GDC) | 1500 ℃ | 1.24×10^-2^ | - | (Lima et al., 2015) |
| Ce_0.8_Gd_0.2_O_2-δ_ | 1200℃ | - | 1.6×10^-2^ | (Anjaneya and Singh, 2017) |
| 3BiGDC | 1200℃ | 2.25×10^-2^ | - | (Accardo et al., 2018) |
| 4Er_0.2_Bi_0.8_O_1.5_-Gd_0.2_Ce_0.8_O_2-δ_ | 1200℃ | 1.03×10^-2^ | 2.69×10^-2^ | (Zhou et al., 2022) |
| NDC | 1200 ℃ | 1.78×10^-3^ | - | (Wang et al., 2019) |
| NDC/MoO_3_ | 1300 ℃ | - | 2.90×10^-2^ | (Xia et al., 2012) |
| BaCe_0.7_Zr_0.1_Y_0.2_O_3-δ_-2%CuO-Bi_2_O_3_ | 1150℃ | 8.5×10^-3^ | - | (Babar et al., 2022) |
| SDC-BZCY/1.5Fe | 1400 ℃ | - | 8.06×10^−3^ | (Ding et al., 2019) |
| BaZr0.8Y0.2O3-δ | 1400 ℃ | - | 6.90×10^−3^ | (Li et al., 2018) |
